# Handmade solar dryer: an environmentally and economically viable alternative for small and medium producers

**DOI:** 10.1038/s41598-021-94353-8

**Published:** 2021-08-25

**Authors:** Wellington Souto Ribeiro, Adriano Sant’Ana Silva, Álvaro Gustavo Ferreira da Silva, Ana Marinho do Nascimento, Marcelo Augusto Rocha Limão, Franciscleudo Bezerra da Costa, Pahlevi Augusto de Souza, Alexandre José de Melo Queiroz, Osvaldo Soares da Silva, Pluvia Oliveira Galdino, Rossana Maria Feitosa de Figueirêdo, Silvanda de Melo Silva, Fernando Luiz Finger

**Affiliations:** 1 Programa de Pós-Graduação em Horticultura Tropical, Universidade Federal de Campina Grande, Pombal, Paraíba 58840-000 Brazil; 2 Unidade Acadêmica de Tecnologia de Alimentos, Universidade Federal de Campina Grande, Pombal, Paraíba 58840-000 Brazil; 3 Universidade Federal da Paraíba, Areia, Paraíba 58397-000 Brazil; 4 Universidade Federal de Viçosa, Viçosa, Minas Gerais 36570-900 Brazil; 5 Instituto Federal do Ceará, Currais Novos, Rio Grande do Norte 63400-000 Brazil; 6 Programa de Pós-Graduação em Engenharia de Processos, Universidade Federal de Campina Grande, Campina Grande, Paraíba 58429-900 Brazil

**Keywords:** Environmental sciences, Agroecology, Applied mathematics

## Abstract

The solar dryer can reduce production costs, energy consumption, waste (use fruits outside the quality standard for fresh consumption) and is an alternative for small and medium producers. The solar dryer can reduce costs and is an alternative for small and medium producers worldwide. The consumption of fresh and processed tomatoes is high in the world, but post-harvest losses is also and drying is an alternative to reduce these losses. The temperature maintenance and drying time corresponds 30% of the costs. The objective was evaluated the tomato physicochemical characteristics after drying in handmade solar dryer. ‘Carmen’ tomato fruits were bleached in water, 2.5% NaCl solution, 2.5% NaCl + 0.5% CaCl_2_ solution and unbleached. Tomato slices were placed in a handmade solar dryer from 7:00 to 17:00. The solar dryer prototype was wood made, comprising a collector and a drying chamber. The average cost of the camera was US$ 13.08 (1 Brazilian Real = 0.26 United States Dollar). Water loss, drying kinetics, mathematical models and physicochemical characteristics of fresh and dried tomatoes were evaluated. The average length of solar drying for the four treatments was 30 h and the Midilli and Kucuk mathematical model was the most adjusted. The acidity, reducing sugars and soluble solids were concentrated by drying, while ascorbic acid was reduced. The pH did not change. Tomatoes 'Carmen' can be dried in a handmade solar dryer for 30 h while maintaining product quality.

## Introduction

Tomato is the second most horticultural crop in the world, with a production of 40 million tons per year^1,2^. The tomato fruits can be consumed in natura and/or processed in the form of dried tomato, powder, juice, puree, concentrated pulp, extract, ketchup and sauces^3^.

Tomato is a climacteric fruit and its post-harvest shelf life is short due to the rapid loss of firmness (softening) during maturation^4^. The main cause of rotting of fruit is the extent of softening that direct effect on palatability, shelf life, resistance to postharvest infections, transportation, storage and consumer acceptance^5,6^.

Drying of agricultural products is one of the most commonly used unitary operations to extend the shelf-life of perishable fruits by reducing water content to safe levels, as well as reducing storage and transport costs^7^. However, convection hot air-drying using fossil fuels such as coal, diesel oil and natural gas generates considerable energy costs and promotes negative environmental impacts^8^. Solar drying is a viable alternative to meet the energy demand, protecting the environment and reducing the energy cost of drying using fossil fuels^9^. In this method, the thermal energy absorbed by the solar collector is supplied to the air inside to dry products to a desired water content^7^.

Drying should still give the final product its own sensory characteristics and preserve its nutritional value to the maximum extent. Pre-treatments are applied to accelerate drying, reducing fruit exposure time and consequent nutritional loss. Bleaching facilitates internal mass transfer during drying by increasing tissue permeability^10^. Salts used in bleaching are also capable of increasing the diffusion of water from the interior of the food to its surface, accelerating drying^11^. Osmotic dehydration caused by salts also preserves the nutritional constituents and color compounds and aroma^12^.

The objective was to evaluate the efficiency of handmade solar dryer for drying tomatoes 'Carmen'.

## Results

The mathematical model of Midilli and Kucuk was the one that most adjusted to the solar drying of tomatoes (Table 1).

**Table 1:** Mathematical models parameters to represent tomato solar drying.

Model	Trat	Parameters				R^2^	DQM
		a	k (min^−1^)	n	b		
Page	T1	–	0.0949	1.0700	–	0.9889	0.0308
	T2	–	0.1148	1.0392	–	0.9871	0.0327
	T3	–	0.1122	1.0897	–	0.9890	0.0415
	T4	–	0.1150	1.0619	–	0.9908	0.0205
Henderson e Pabis	T1	1.0348	0.1161	–	–	0.9943	0.0312
	T2	1.0347	0.1299	–	–	0.9879	0.0318
	T3	1.0531	0.1436	–	–	0.9894	0.0300
	T4	1.0445	0.1372	–	–	0.9913	0.0270
Midilli e Kucuk	T1	1.0342	0.1260	0.9241	− 0.0021	0.9919	0.0263
	T2	1.0424	0.1425	0.9436	− 0.0009	0.9886	0.0307
	T3	1.0405	0.1334	1.0213	− 0.0003	0.9899	0.0293
	T4	1.0374	0.1384	0.9817	− 0.0007	0.9919	0.0260

*T1* no bleaching, *T2* bleaching, *T3* bleaching in 2.5% NaCl solution, *T4* bleaching in 2.5% NaCl and 0.5% CaCl_2_ solution.

**Figure 1:**
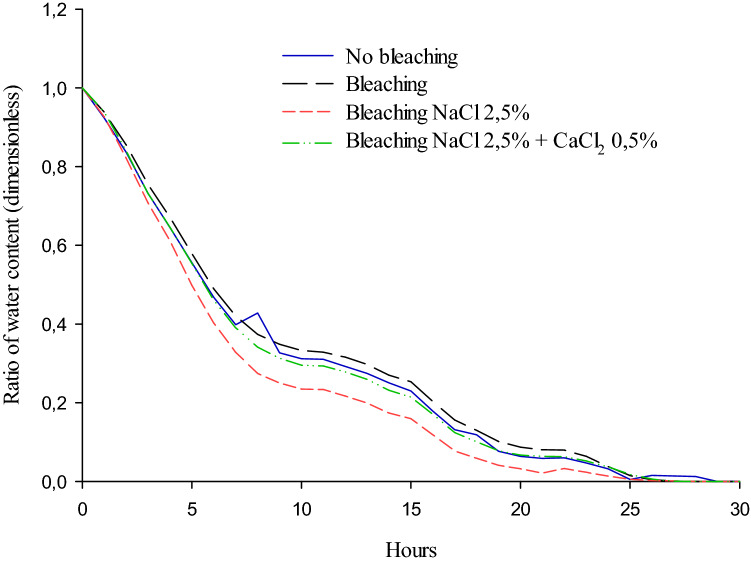
Drying kinetics curves of the tomato 'Carmen' using handmade solar dryer and direct exposure to solar collector.

**Figure 2:**
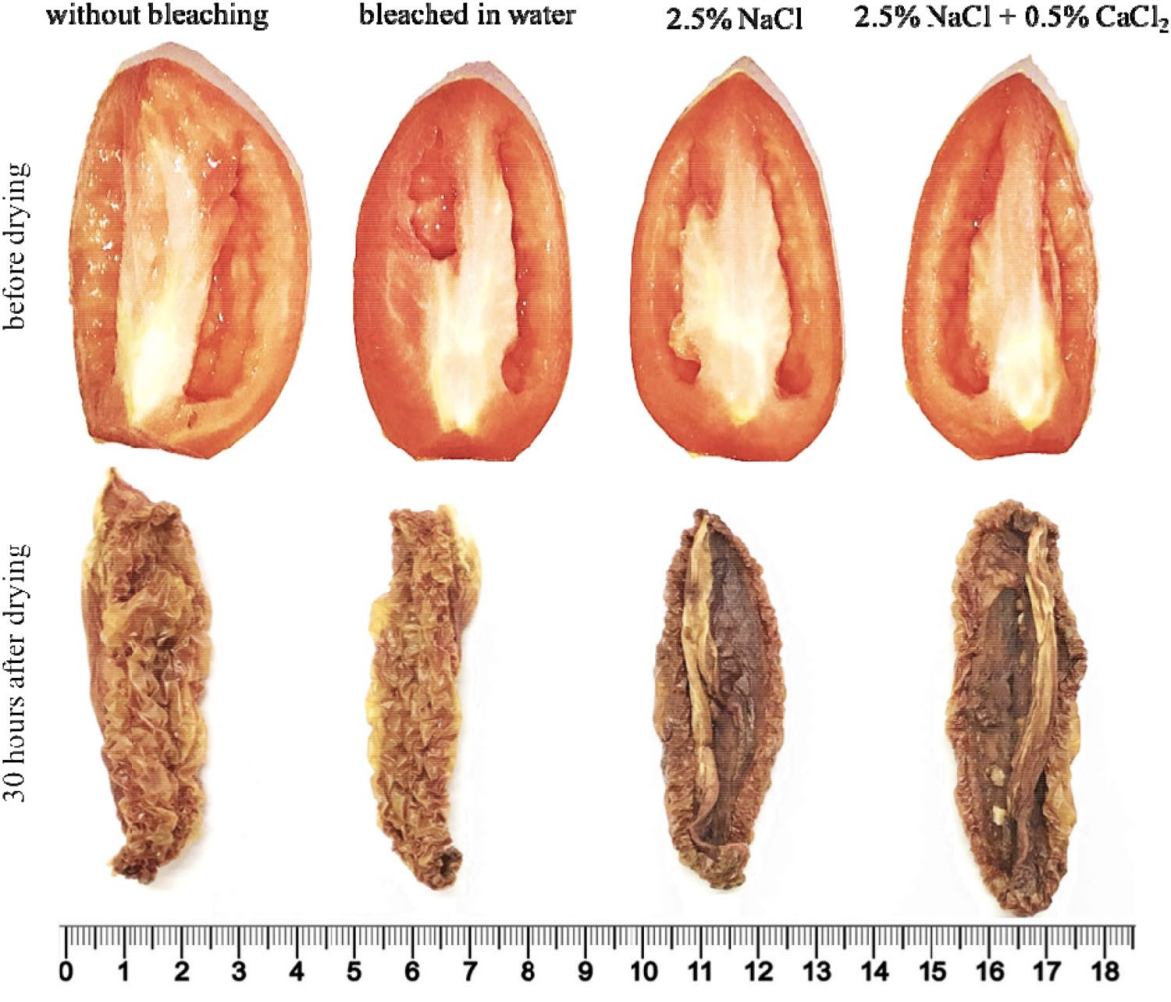
General view of 'Carmen’ tomatoes pre-treated before drying and 70 h after drying. Scale in centimeters. Source: authors.

The drying rate of sliced tomatoes did not differ between treatments, was higher in the first 10 h and progressively reduced until the end of the process. The mean drying time was 30 h and did not differ between treatments. The dryer temperature in the solar dryer varied from 40 (07:00–09:00 and 17:00) to 70 °C (09:00–16:00) (Figs. 1 and 2).

The water content (WC) in slices of pre-treated tomatoes with bleaching in 2.5% NaCl solution and 2.5% NaCl solution and 0.5% CaCl_2_ was lower after drying. Titratable acidity (TA), soluble solids (SS) and reducing sugars (RS) increased with drying and did not differ between treatments. The ascorbic acid content (AsA) decreased with drying and did not differ between treatments. The pH was not changed upon drying (Table 2).

**Table 2:** Physical–chemical characterization of fresh and dried tomatoes in a handmade solar dryer.

Trat	WC	TA	pH	SS	AsA	RS
In natura	95.7 a ± 2.1	0.7 b ± 0.2	4.4 a ± 0.0	3.8 b ± 0.0	26.6 a ± 3.9	0.31 a ± 0.02
T1	30.8 b ± 2.1	2.9 a ± 0.6	4.4 a ± 0.0	45.7 a ± 0.6	17.9 b ± 1.0	4.31 b ± 0.81
T2	30.0 b ± 2.1	3.5 a ± 0.5	4.4 a ± 0.0	48.3 a ± 0.6	20.5 b ± 0.5	4.44 b ± 0.65
T3	25.6 c ± 2.1	3.9 a ± 0.4	4.4 a ± 0.0	49.3 a ± 1.2	21.6 b ± 1.5	4.84 b ± 0.70
T4	24.4 c ± 2.1	3.5 a ± 0.8	4.4 a ± 0.0	47.0 a ± 1.7	19.7 b ± 1.0	4.72 b ± 0.66

Means followed by the same letter in the columns did not differ by the Tukey test (p < 0.05).

*T1* no bleaching, *T2* bleaching in water, *T3* bleaching in 2.5% NaCl solution, *T4* bleaching in 2.5% NaCl and 0.5% CaCl_2_, *WC* water content (%), *TA* titratable total acidity (% citric acid), *SS* total soluble solids, *AsA* ascorbic acid (mg 100 g^−1^), *RS* reducing sugars (%).

## Discussion

All applied models represented precisely the process, with R^2^ above 99%. Nevertheless, the model of Midilli and Kucuk fit better among the others.

The drying rate in the sliced tomatoes was higher at the beginning of the process due to the higher absorption of radiation by the high water content. Mature tomato fruits, variety regardless, contain, on average, 93–95% water^13^. Water is a good heat transfer fluid because it has high thermal capacity and low viscosity, accelerating drying^14^. The gradual reduction in the drying rate after 10 h is explained by the reduction in the water content during the process. The average time of 30 h of drying at 40–70 °C was satisfactory, since there were no costs in the energy consumption and the dry product quality was adequate. The optimal dehydration conditions for tomato slices are 35–44 h at 52–67 °C^15^. The maintenance of temperature and drying time correspond to 30% of the total cost of processing^16^, since fruits and vegetables generally contain more than 80% water and the drying process at desirable levels (5–10%) consumes a lot of energy^16^. Considering the low set-up cost, zero energy cost, reduced spatial impact^17^, favorable public acceptance^18,19^ and obtaining a product with acceptable qualities, the handmade solar dryer is an environmentally and socioeconomically beneficial system that characterizes the activity as an innovative business model for small and medium producers^17^.

The lower WC in the slices of pretreated tomatoes with bleaching in 2.5% NaCl solution and 2.5% NaCl and 0.5% CaCl_2_ after drying can be explained by the osmotic dehydration caused by the salt presence. In this phenomenon, water was removed from the inner tissues of the tomato slices (lower concentration of solute) to the surface (higher concentration) through semipermeable membranes to maintain equilibrium on both sides of the membrane^20^. On the surface, the water absorbed greater radiation accelerating the drying^14^ and resulting in products with lower WC. The increase of TA, SS and RS is basically explained by the concentration of organic acids and soluble solids due to the water loss. The AsA content reduced with drying, regardless of the treatment, due to the reduction in water content in tomato slices. The AsA loss during the tomato drying process depends on the temperature, drying time, product humidity^21^, luminosity and pH^22^ and follows a first order kinetics. This means that AsA degradation during tomato drying is a result of increased tomato concentration increasing the degradation rate^23^. The AsA initial rate degradation was faster as the moisture content decreased^24^. The presence of salts in the bleaching may also have influenced the AsA degradation. AsA was diffused from internal tissues to the tomato slices surface where chemical degradation occurred^25^. AsA photooxidation occurs in water at pH 4.5–11.6^22^ similar to those found in this experiment.

## Conclusions

The model of Midilli and Kucuk was the one that best represented the drying process, with R^2^ superior to 99%. ‘Carmen’ tomatoes can be dried with handcrafted solar dryer for 30 h while maintaining product quality.

## Material and methods

### Obtaining and processing raw material

Fruits tomatoes 'Carmen' were purchased in the municipal market Pombal, Paraíba, Brazil, selected in advanced stage of maturation (very mature) and taken to the laboratory. In the laboratory, the fruits were washed in double-filtered running water, sanitized in chlorinated solution at 100 ppm for 15 min, rinsed, sliced manually in four equal parts and the seeds were removed. One hundred grams of the sliced fruits were bleached in water (T2), 2.5% NaCl solution (T3), 2.5% NaCl solution and 0.5% CaCl_2_ (T4) or without bleaching (T1). Bleaching occurred at 100 °C for 2.5 min with immediate immersion in ice water (0 ± 4 °C).

### Solar drying

**Figure 3:**
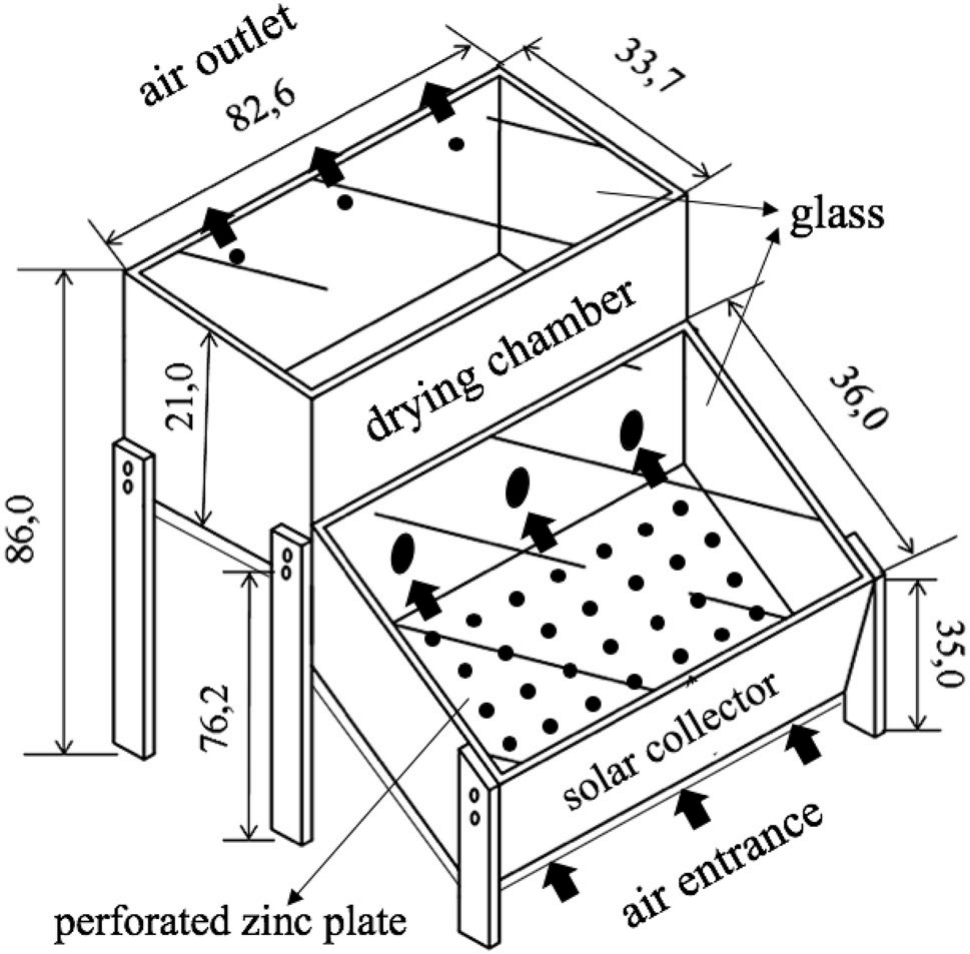
Handmade solar dryer and direct exposure to solar collector used in drying tomatoes 'Carmen'. Dimensions expressed in centimeters. Source: authors.

Drying was conducted in a homemade solar dryer direct exposure to light collector and 0.32 m^2^ drying chamber. The equipment was made in plywood in black. The collector and drying chamber were covered with 2 mm glass plates. The heat sink body of the solar collector was black zinc (Fig. 3).

The tomato slices, bleached or not, were randomly placed in the solar desiccator from 7:00 a.m. to 5:00 p.m. for 3 days, totaling 30 h of sun exposure. The slices were weighed every hour and drying was completed after obtaining constant weight over three consecutive weighings. The dried slices were packed in high density polypropylene, vacuum sealed, covered with aluminum foil and stored in a refrigerator. This process was performed in triplicate and on different days. Water loss, drying kinetics and mathematical models were evaluated. The moment of the removal of the solar dryer treatments was calculated:

1 $${\text{Mf}} = {\text{Mi}} \times \left( {100 - {\text{MOi}}} \right)/\left( {100 - {\text{MOf}}} \right)$$

where, Pi is the initial mass of the tomato (g), Mf is the final mass of tomato after dry (g), MOi is the initial moisture of fresh tomato and MOf is the desired final moisture of dried tomato (25–30%).

### Analysis of the drying kinetics

The semi-theoretical mathematical models of Page, Henderson and Pabis and Midilli and Kucuk^26^ were applied to evaluate which was the most representative for drying (Table 3).

**Table 3:** Mathematical models used in the analysis of the solar drying process.

Models	Equation
Page	WCR = exp (− k∙t^n^ )
Henderson e Pabis	WCR = a∙exp (− k∙t)
Midilli e Kucuk	WCR = a∙exp (− k∙t^n^ ) + b∙t

WCR is the water content ratio (WCR = (U_t _− U_eq_)/(U_0 _− U_eq_), k is the drying constant, b e n is the parameters of the models, t is the process time.

The models were applied by non-linear regressions using Statistica software version 5.0. The highest coefficient of determination (R^2^) and the lowest mean square deviation (MSD) (Eq. 2) were used as parameters for model evaluation^27^. Equation (2):

2 $${\text{MSD}} = \surd \left( {\Sigma \left( {{\text{RTAexp}} - {\text{WCRpre}}} \right)^{{2}} /{\text{N}}} \right),$$

RTA_exp_ is the experimental water content ratio, WCR_pre_ is the ratio of water content predicted by equation, N is the observations number made during the experiment.

### Physicochemical analysis

Fresh and dried tomatoes were evaluated:

- *Total titratable acidity* five grams of pulp were homogenized in 45 mL of distilled water and the solution was titrated with sodium hydroxide (0.1 N) to pink color^28^;
- *pH* determined by potentiometer reading (Tecnal, TEC-2);
- *Reducing sugar* the reducing sugars content was determined by the dinitro alicyclic acid method^29^. An extract was prepared by diluting 1.0 g of pulp in 100 ml of distilled water. A solution containing 0.5 ml of the extract, 1.0 ml of distilled water and 1.0 ml of the dinitrosalicyclic acid solution was prepared in tubes. The tubes were agitated rapidly in a stirrer (Novainstruments, NI 1107, Piracicaba, São Paulo, Brazil) and placed in a thermostatic bath (Hemoquímica, HM 0128, Sabará, Minas Gerais, Brazil) at 100 °C for 5 min. The reducing sugars content was determined by spectrophotometry (Spectrum, 560 UV, Maharashtra, India) at 540 nm using glucose as reference for obtaining the standard curve.
- *Water content* sliced tomato samples were spread separately on trays and dried by heat dehydration in an electric oven (SOLAB) at 70 °C to constant mass^28^;
- *Soluble solids* drops of juice from fruit pulp pressed was placed on the digital refractometer prism and soluble solids was determinate by ^o^Brix (Digital Refractometer, New Jersey, USA);
- *Ascorbic acid* five grams of pulp were homogenized in 47 ml of oxalic acid (0.5%) and titrated with Tillmans solution until pink^28^.

### Statistical analysis

The results of the physicochemical evaluation were submitted to Analysis of Variance (ANOVA) and the means were compared by the Tukey test at 5% significance by the software Assistat, version 7.7^30^.
